# A comparative evaluation of calix[4]arene-1,3-crown-6 as a ligand for selected divalent cations of radiopharmaceutical interest†

**DOI:** 10.1039/c9ra07293d

**Published:** 2019-10-10

**Authors:** David Bauer, Markus Blumberg, Martin Köckerling, Constantin Mamat

**Affiliations:** Institut für Radiopharmazeutische Krebsforschung, Helmholtz-Zentrum Dresden-Rossendorf Bautzner Landstraße 400 D-013228 Dresden Germany c.mamat@hzdr.de; Fakultät Chemie und Lebensmittelchemie, TU Dresden D-01062 Dresden Germany; Institut für Chemie-Anorganische Festkörperchemie, Universität Rostock Albert-Einstein-Straße 3a D-18059 Rostock Germany

## Abstract

Metals, which form divalent cations, including the alkaline earth metals offer radionuclides like lead-203, lead-212, barium-131, and strontium-89, which are promising candidates for radiopharmaceutical applications. Besides, the heavy homologous nuclides radium-223 and radium-224 – with similar properties to barium – are suitable alpha-emitters for targeted alpha-particle therapy. However, there is a lack of suitable chelation agents, especially for heavy group 2 metals. The macrocycle calix[4]arene-1,3-crown-6 seems to interact with these metals strongly. Therefore, this ligand and its coordination to the divalent cations of barium, strontium, and lead have been investigated. The complex formation was analyzed by NMR and UV/Vis titration experiments in acetonitrile, and stability constants were determined to be >4 with both methods. It was found that the stability of these complexes increase in the order strontium, barium, and lead. Additional to these investigations, X-ray crystallography, solvent-dependent ^1^H NMR, and ^207^Pb NMR measurements were performed to deliver deeper insight into the coordination chemistry of this ligand.

## Abstract 2

The stability constants of the promising ligand calix[4]arene-1,3-crown-6 and divalent metals of radiopharmaceutical interest: lead, barium, and strontium, were determined *via* NMR and UV/Vis techniques.

## Graphical abstract

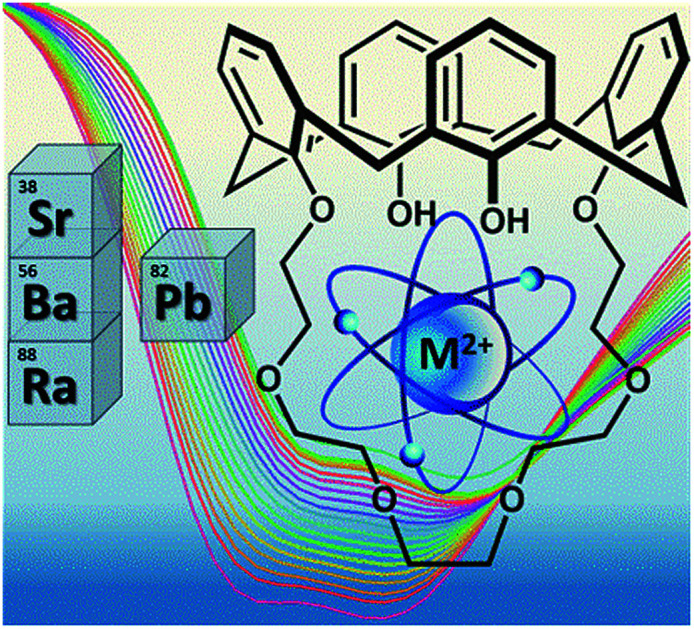

## Introduction

Nuclear medicine is a strongly growing, active area of academic and commercial research based on the application of radionuclides for diagnostic and therapeutic purposes. The potential of radiometals has more recently been realized and relies on their nature of being easily attached to a biomolecule by implementing a chelating agent.^1–3^ The development of selective and specific chelators is, for this reason, a pivotal aspect in expanding the clinical capabilities of selected radiometals.

For this work, the divalent cations Sr^2+^, Ba^2+^, and Pb^2+^ have been selected, since they provide radionuclides, which possess useful properties for application in radiopharmacy as well as nuclear medicine and are therefore of particular interest.

In the field of targeted and pre-targeted radioimmunotherapy, ^212^Pb has demonstrated significant utility in both *in vitro* and *in vivo* systems.^4^ The radionuclide ^212^Pb is not only a promising β^−^-emitter but serves as an *in vivo* generator for the alpha-emitter ^212^Bi.^5^ Alpha-emitters combine a short range with a high linear energy transfer, which results in the relatively high biological effect and cytotoxicity.^6^ Furthermore, the combination of ^212^Pb and ^203^Pb is a matched radionuclide pair for image-guided radionuclide therapy. ^203^Pb decays by electron capture with a γ-emission of 279 keV (80%) which makes it a promising SPECT (single photon emission computer tomography) imaging agent.^7^

Alkaline earth metals belong to the most prominent group of divalent ions, which offer a variety of interesting radionuclides. The utility of ^89^Sr in the treatment of advanced metastatic prostate cancer has been examined in numerous clinical trials.^8–11^^89^Sr in the form of its dichloride (Metastron) is FDA-approved for radionuclide therapy since 1993 and is used for the palliation of painful osseous metastases.^12^ The element barium offers the radionuclide ^131^Ba, which decays by electron capture while emitting suitable γ-rays for diagnostic use. The radionuclide ^131^Ba could be a promising bone-scanning agent in scintigraphy.^13,14^ The group of heavy alkaline earth metals can also be expanded to its heaviest homolog. The element radium offers the α-emitters ^223^Ra and ^224^Ra. Due to their suitable half-lives of 11.4 d, respectively 3.6 d, and their excellent decay properties (a cascade of 4 × α and 2 × β decays), both are ideal nuclides for the targeted alpha-particle therapy.^15^ Radium, in the form of [^223^Ra]RaCl_2_ (Xofigo), is indicated for the treatment of metastatic castration-resistant prostate cancer (mCRPC) and received marketing approval by the EMA and FDA in 2013.^16^ Since there is no stable radium isotope, it is not a trivial challenge to investigate its chemistry. However, due to its related chemical behavior, barium can be seen as its non-radioactive surrogate and findings derived from the barium chemistry will most likely apply for radium as well.^17–19^ All mentioned radionuclides and their properties are listed in Table 1.

**Table 1:** Properties of selected radiometal nuclides.^21–26^ The ionic radii were reported in the literature for the coordination number VIII and the charge state 2+ (ref. 27)

Nuclide	Radii (Å)	*t*_1/2_	Decay mode	*E* (keV)	Production method	Comments	Application
^203^Pb	1.29	51.9 h	ε (100%)	279 (γ)	^203^Tl(p,n)^203^Pb		SPECT
^212^Pb		10.6 h	β^−^ (100%)	570	^224^Ra/^212^Pb generator	Daughter is ^212^Bi (α)	β^−^/α therapy
^89^Sr	1.26	50.6 d	β^−^ (100%)	1.5 × 10^3^	^88^Sr(n,γ)^89^Sr		β^−^ therapy
^131^Ba	1.42	11.5 d	ε (100%)	124, 216 (γ)	^131^Cs(p,n)^131^Ba		SPECT
^223^Ra	1.48	11.4 d	α (100%)	6.0 × 10^3^	^227^Ac/^223^Ra generator	Decay chain with 4 × α and 2 × β	α therapy
^224^Ra		3.6 d	α (100%)	5.8 × 10^3^	^228^Th/^224^Ra generator		

Although these nuclides possess suitable properties, the absence of stable bifunctional chelating agents for these radiometals hampers their use for targeted radiotherapy and radioimaging.^1^ The essential requirement for a good chelator is the thermodynamic and kinetic stability of its metal–chelate complex *in vivo*; this necessitates the design of ligands appropriate for the particular radionuclide.^2,20^ To compare and evaluate chelators with high complexation ability, the knowledge of the stability constant (log *K*) is mandatory.

There is a constant search for ligands perfectly adapted to the particular application. Noteworthy, the recently developed DOTA-derivative TCMC (1,4,7,10-tetrakis(carbamoylmethyl)-1,4,7,10-tetraazacyclododecane) seems to be promising for Pb.^28,29^ Though, there are no remotely suitable chelating agents known for heavy alkaline earth metals.

Calix-crown ethers are a widely investigated class of ligands for cations based on the calixarene backbone. They provide a suitable coordination chemistry towards alkali metal and alkaline earth metal cations.^30–34^ Depending on the metal, the coordination appears to involve not just the crown ether-oxygen donors, but also the aromatic phenol-units of the calixarene.^35^^1^H NMR studies can provide further experimental evidence for the influence of cation–π-interactions and are a key part of this work.

The calix[4]arene-1,3-crown-6 bears two hydroxyl groups located on opposite sides. These groups act as ionizable units by the loss of their protons under basic conditions and thus to form a neutral complex witch a divalent cation. Additionally, the hydroxyl functions can straightforwardly be modified by attaching other ionizable units.

The objective of this research was to evaluate calix[4]arene-1,3-crown-6 (1) as a lead compound that could, upon further modifications, yield a viable ligand for the above mentioned divalent cations. Literature about alkaline earth metal ligands, especially about radium, are mostly focused on extraction studies without information about comparable stability constants.^32,36,37^ Therefore, UV/Vis and NMR titration experiments were established as reliable and constant methods for the determination of the stability constants of calix 1 with Sr^2+^, Ba^2+^, and Pb^2+^. Due to an activity-related concentration limit, Ra^2+^ could not be investigated.

## Results and discussion

### Synthesis and characterization of ligand 1

Two synthetic routes are described in the literature for the preparation of ligand 1, both using the starting material calix[4]arene. A high yield of the ligand 1 can be obtained when first alkylating the calix[4]arene to obtain the 1,3-dimethylcalix[4]arene, followed by the alkylation of the remaining two hydroxyl groups with pentaethylene glycol ditosylate (crown ether moiety) followed by the cleavage of the methyl groups with trimethylsilyl iodide.^31^

The second route is a one-pot reaction starting directly with the calix[4]arene and pentaethylene glycol ditosylate. Potassium *tert*-butoxide is used as a base to deprotonate the hydroxyl groups; additionally the potassium cations serve the purpose of a template effect and arrange the formation of the cone conformation. However, this reaction takes 3 days and obtains a moderate yield.^38^ For this work, ligand 1 was obtained with a higher yield of 50% using a modified reaction according to the second route.^39^

**Fig. 1:**
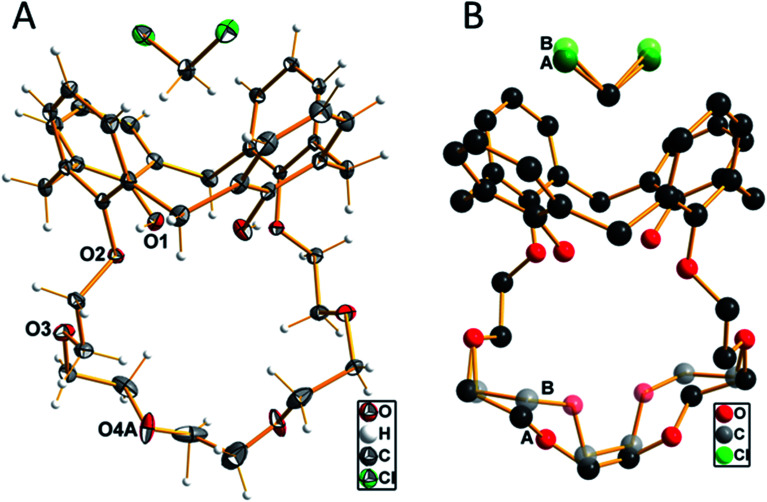
(A) Molecular structure of compound 1 with the labeling of the oxygen atoms. Displacement ellipsoids are shown at the 50% probability level at −150 °C. Only one of the two orientations of the disordered parts of the ether chain and the dichloromethane molecules are shown. (B) View of the disorder in half of the crown ether chain and the dichloromethane molecule, indicated by letters A and B and color differentiation.

When slowly evaporating the solvent of a saturated solution of 1 in dichloromethane, the formation of single crystals was observed. These crystals were analyzed by single-crystal X-ray diffraction, which unfolded an inclusion complex with dichloromethane located in the upper rim of the aromatic calix[4]arene part (Fig. 1). Compound 1 crystallizes in the monoclinic crystal system with the centrosymmetric space group *P*2/*n*. The *n* glide plane of the space group cuts through the calixarene molecule as well as through the carbon atom of the dichloromethane molecule. Therefore, the asymmetric unit contains only half of both molecules. The part of the crown ether ring starting from O3 up to O3′ ( <svg xmlns="http://www.w3.org/2000/svg" version="1.0" width="18.545455pt" height="16.000000pt" viewBox="0 0 18.545455 16.000000" preserveAspectRatio="xMidYMid meet"><metadata> Created by potrace 1.16, written by Peter Selinger 2001-2019 </metadata><g transform="translate(1.000000,15.000000) scale(0.015909,-0.015909)" fill="currentColor" stroke="none"><path d="M80 840 l0 -40 -40 0 -40 0 0 -40 0 -40 40 0 40 0 0 40 0 40 120 0 120 0 0 -40 0 -40 -80 0 -80 0 0 -40 0 -40 80 0 80 0 0 -80 0 -80 -120 0 -120 0 0 40 0 40 -40 0 -40 0 0 -40 0 -40 40 0 40 0 0 -40 0 -40 120 0 120 0 0 40 0 40 40 0 40 0 0 80 0 80 -40 0 -40 0 0 40 0 40 40 0 40 0 0 40 0 40 -40 0 -40 0 0 40 0 40 -120 0 -120 0 0 -40z M720 840 l0 -40 -40 0 -40 0 0 -80 0 -80 -40 0 -40 0 0 -80 0 -80 -40 0 -40 0 0 -80 0 -80 -40 0 -40 0 0 -80 0 -80 -40 0 -40 0 0 -40 0 -40 -40 0 -40 0 0 -40 0 -40 80 0 80 0 0 40 0 40 40 0 40 0 0 80 0 80 40 0 40 0 0 80 0 80 40 0 40 0 0 -40 0 -40 80 0 80 0 0 40 0 40 40 0 40 0 0 -80 0 -80 -40 0 -40 0 0 -40 0 -40 -40 0 -40 0 0 -40 0 -40 -40 0 -40 0 0 -40 0 -40 200 0 200 0 0 40 0 40 -80 0 -80 0 0 40 0 40 40 0 40 0 0 80 0 80 40 0 40 0 0 40 0 40 -40 0 -40 0 0 40 0 40 -120 0 -120 0 0 -40 0 -40 -40 0 -40 0 0 80 0 80 40 0 40 0 0 80 0 80 40 0 40 0 0 80 0 80 -40 0 -40 0 0 -40z"/></g></svg> − *x*, *y*, ½ − *z*) is disordered with two different orientations as well as the chlorine atom of the dichloromethane molecule. This disorder is refined using split positions (A and B) for each affected atom (group). The different orientations of the ether part and the dichloromethane molecule are shown in Fig. 1B.

The four aromatic rings of the calixarene form a cavity, which is expanded at the upper rim. The opposite rings are inclined to each other with an angle of 34.5° of the two rings, which are attached to the crown ether moiety, compared to the other two unsubstituted rings, where the angle between the two main planes through the ring carbon atoms was determined with 84.2°. A little above the upper rim, the (disordered) dichloromethane molecule is located. The two hydrogen atoms, whose positions were initially located from the difference Fourier map and later refined using riding models, are oriented such that they have relatively close distances to the centers of the phenyl groups, which are attached to the crown ether residue. The distance of these protons to the center of the rings is 2.485 Å. This is close enough to be discussed as weak C–H–π interaction.^40^ The oxygen atoms of the crown ether moiety (O2, O3, O4(A, B) and symmetry equivalents) are not primarily directed toward the inner part of the cavity, as they would do whenever a metal cation is coordinated. The diameter of this cavity, as defined by the distances of opposite oxygen atoms, is 5.887 Å (O2–O4A′ (−½ + *x*, 1 − *y*, ½ + *z*) 2 times) and 6.849 Å (O3–O3′ (−½ + *x*, 1 − *y*, ½ + *z*)). The shortest hydrogen bonds are found intermolecularly as O1–H1A⋯O2 contact at a D⋯A distance of 2.767(1) Å and a D–H⋯A angle of 167.7°. All other possible proton contacts have D⋯A distance longer than 3.0 Å.

### Initial ^1^H NMR studies

**Fig. 2:**
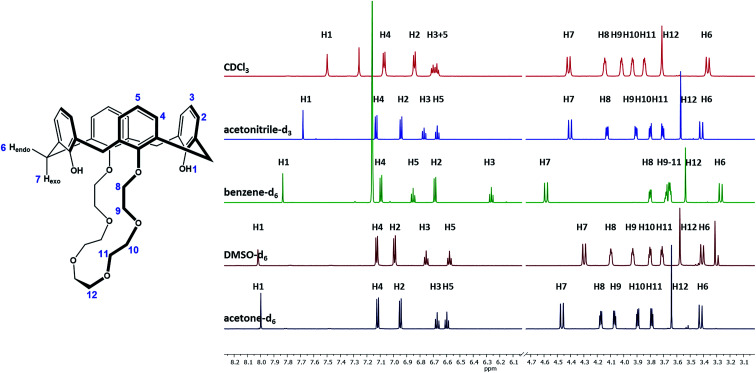
^1^H NMR spectra of ligand 1 in different solvents.

Due to the results derived from the X-ray spectroscopy, it had to be assumed that the solvent has a strong influence not only on the solid-state conformation but also on the conformation of ligand 1 in solution. Since the incorporation of small molecules and solvent molecules into calixarenes has been widely discussed in the literature in general,^41–43^ but not particularly for ligand 1, it was necessary to investigate the influence of the solvent on its conformation. Due to their diversion in polarity, size and solvation effects, the solvents CDCl_3_, acetonitrile-d_3_, benzene-d_6_, DMSO-d_6_, and acetone-d_6_ have been selected, and the interaction with ligand 1 was studied by ^1^H NMR measurements (Fig. 2). The same amount of 1 was dissolved in 0.7 mL of the respective solvent and measured at 298 K using a 400 MHz NMR spectrometer. All proton signals have been assigned and listed in Table 2. Separated signals for the crown ether and benzylic protons were found, indicating that the presence of intramolecular hydrogen bonds stabilizes the conformation. The fact that strong hydrogen bonds are present can also be derived from the H1 hydroxyl signals, since their sharp contour evidence a strong intramolecular hydrogen binding.^44^ These stabilization effects, causing a rigidification of the host molecule, have already been reported in the literature.^45^ A *C*_2_-symmetry is found for ligand 1, which results in the number of 12 signals in the ^1^H NMR spectrum. For all spectra measured in the different solvents, two signals for the methylene bridge are found (endo H6, exo H7), proving the cone conformation of the calix crown (signals would coincide if a phenolic ring flips).^45^ The distance between H6 and H7 indicates the angle of the aromatic rings. If the rings are facing each other, the Δ*δ*_H7–H6_ value should be in the range of 0.9 ± 0.2 ppm,^45^ which applies to all spectra, but not to benzene (Δ*δ*_H7–H6_ = 1.3).

**Table 2:** ^1^H NMR (400 MHz) shifts of compound 1

^1^H signal^a^	Multiplicity [Hz]	Integral	Shift [ppm]					Assignment
			CDCl_3_	CD_3_CN	C_6_D_6_	(CD_3_)_2_SO	(CD_3_)_2_CO	
H1	s	2	7.50	7.68	7.83	8.02	8.00	OH
H2	d^#^	4	6.84	6.94	6.69	6.99	6.67	Ar–H
H3	t^#^	2	6.73–6.67	6.77	6.26	6.75	6.95	Ar–H
H4	d^§^	4	7.07	7.13	7.09	7.13	7.12	Ar–H
H5	t^§^	2	6.67–6.63	6.67	6.85	6.58	6.60	Ar–H
H6	d*	4	3.37	3.42	3.27	3.41	3.42	H_endo_
H7	d*	4	4.42	4.40	4.58	4.30	4.47	H_exo_
H8	m	4	4.18–4.12	4.14–4.11	3.82–3.78	4.10–4.06	4.20–4.15	CH_2_O
H9	m	4	4.04–4.00	3.93–3.89	3.70–3.63	3.98–3.94	4.09–4.05	CH_2_O
H10	m	4	3.96–3.91	3.82–3.78	3.70–3.63	3.81–3.77	3.92–3.87	CH_2_O
H11	m	4	3.87–3.83	3.73–3.68	3.70–3.63	3.74–3.70	3.80–3.76	CH_2_O
H12	s	4	3.71	3.57	3.54	3.58	3.64	CH_2_O
^#2^ *J* [Hz] =			7.6	7.6	7.6	7.6	7.5	
^§2^ *J* [Hz] =			7.5	7.5	7.5	7.5	7.5	
*^2^*J* [Hz] =			13.1	13.1	13.1	12.8	13.0	

a Is not representing the IUPAC assignment. Compare the assignment with Fig. 2.

**Fig. 3:**
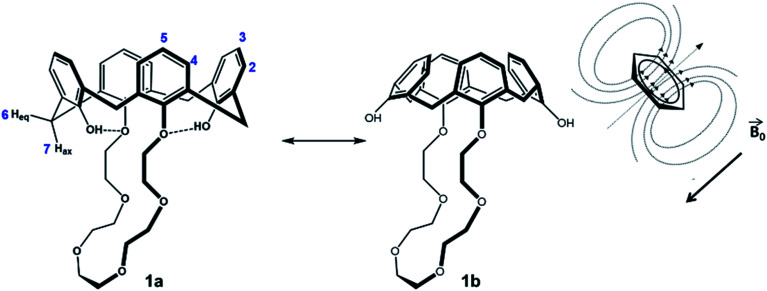
Conformational change of calix 1 and its consequence for the NMR-induced magnetic field.

No significant influence of the solvent was observed when comparing the coupling constants from Table 2 (^2^*J*), and except of benzene-d_6_, the signals in the spectra are similar arranged for the different solvents. As expected, there is an influence of the solvent on the hydroxyls (H1), depending on its polarity and amount of acceptor sites. The stronger the hydroxyl oxygen can coordinate to the solvent molecules, the less electron density remains at the H1 proton (downfield shift). However, this does not apply for the solvent benzene, which has the lowest polarity in comparison, but a significant H1-downfield shift. A likely explanation for the solvent effect of benzene on the calix 1 is illustrated in Fig. 3. The solvent interacts strongly with the phenolic rings (π–π-interaction), which will cause a flattening of the cone (Fig. 3-1b) and a contraction of the upper-rim of the calix 1. Hence, the intramolecular hydrogen bonds break and the H1 signal shifts downfield. The protons H2 and H3 are facing into the cavity. Due to the aromatic ring current effect induced by the magnetic field of the NMR spectrometer, these protons perceive a lower magnetic field, and this will be noticed as a highfield shift in the spectrum. Since the aromatic rings are facing the crown ether, the closest protons (H8–H10) will also perceive a lower magnetic field (highfield shift of their signals).

The other smaller solvents, as represented in the crystal structure (Fig. 1), instead form an inclusion complex with calix 1, which leads to an expansion of the upper rim (Fig. 3-1a).

Comparing the different spectra, the signals received from the acetonitrile-measurement, are sharp and well separated, and there does not seem to be significant interaction with the hydroxyl functions. Additionally, we use metal perchlorate salts to study the ligand–metal-interaction; those are highly soluble in acetonitrile. The use of acetonitrile-d_3_ is superior over DMSO-d_6_, because DMSO is known to form complexes with divalent cations.^39,46^ For these reasons and since the compound is not soluble in water, the stability constants are determined in acetonitrile. Two different methods have been used to determine the stability constants: NMR and UV/Vis titration with the respective metal perchlorate salts.

### NMR titration studies

**Fig. 4:**
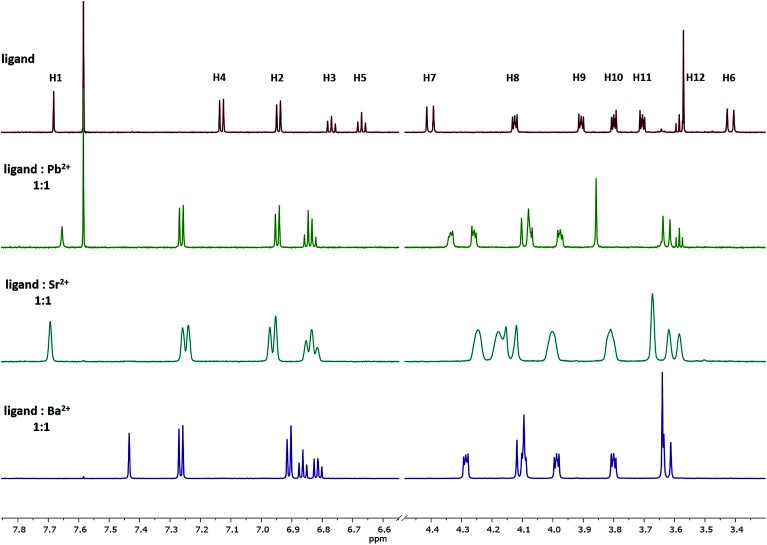
^1^H NMR spectra of the 1:1 complex of ligand 1 with Pb^2+^, Sr^2+^, and Ba^2+^ in acetonitrile-d_3_. The signal at 7.58 represent residual CHCl_3_ and do not affect the method.

Upon the addition of the different metal perchlorate salts, shifts in the ^1^H NMR signals were observed, until the 1:1-complex was completely formed. When comparing the ^1^H NMR spectra of Ba^2+^-, Sr^2+^-, and Pb^2+^-1:1-complexes (Fig. 4), significant shifts for the signals of the protons H8–H12 were determined, representing a strong interaction of the crown ether with the cations. Further, the Δ*δ*_H7–H6_ value shrunk from 1.0 (ligand) to 0.5 ppm (1:1-complex), caused by the contraction of the lower rim (compare with Fig. 3-1a) and indicating the coordination of all four phenolic oxygen atoms to the metal center. It seems that the affinity towards the bivalent cations mainly results from the combination of the calixarene backbone and the crown ether.

**Fig. 5:**
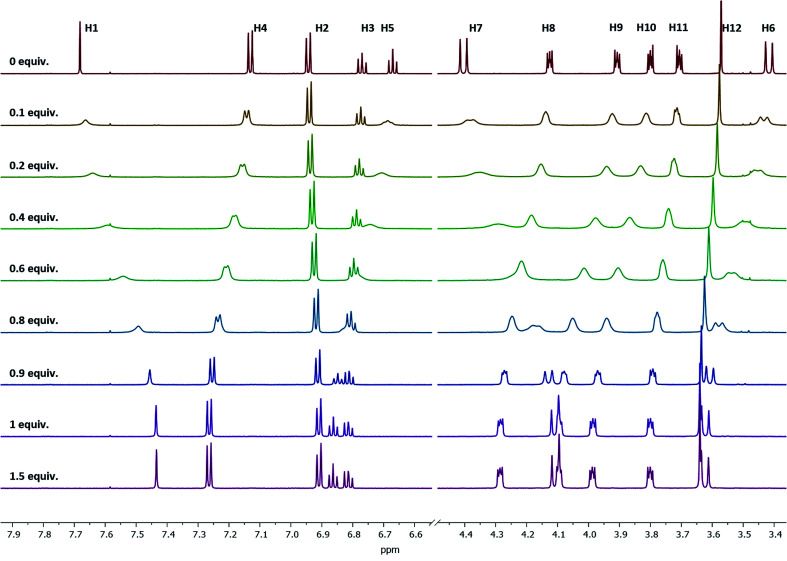
^1^H NMR spectra of ligand 1 at different Ba(ClO_4_)_2_ concentrations measured in acetonitrile-d_3_. Signal H4 is sufficiently pronounced to be used for the stability constant calculations.

Stability constants of these complexes were determined by the relationship between the shifts of a selected proton signal and the equivalents of the cation. A reliable method to determine the stability constants *via*^1^H NMR spectroscopy was developed in the past,^39^ using Pb^2+^, Sr^2+^, and Ba^2+^ as their perchlorate salts and acetonitrile-d_3_ as the solvent. For the calculation of the stability constants, the chemical shift has to be precisely determined. To ensure this, the changes in the chemical shifts of the ^1^H signals between ligand 1 and the cation-containing complexes Pb-1, Sr-1, and Ba-1 must be sufficiently pronounced. Considering all ^1^H NMR signals and their shifts, especially H4 is a suitable signal for these calculations, since it remains sharp and does not overlap with other signals upon complexation. This signal is shifted downfield, as the H4 proton moves out of the aromatic ring current zone caused by the contraction of the lower rim. The dependence of chemical shifts on the metal concentration is illustrated in Fig. 5 by the example of Ba^2+^ (see ESI† for Sr^2+^and Pb^2+^).

**Fig. 6:**
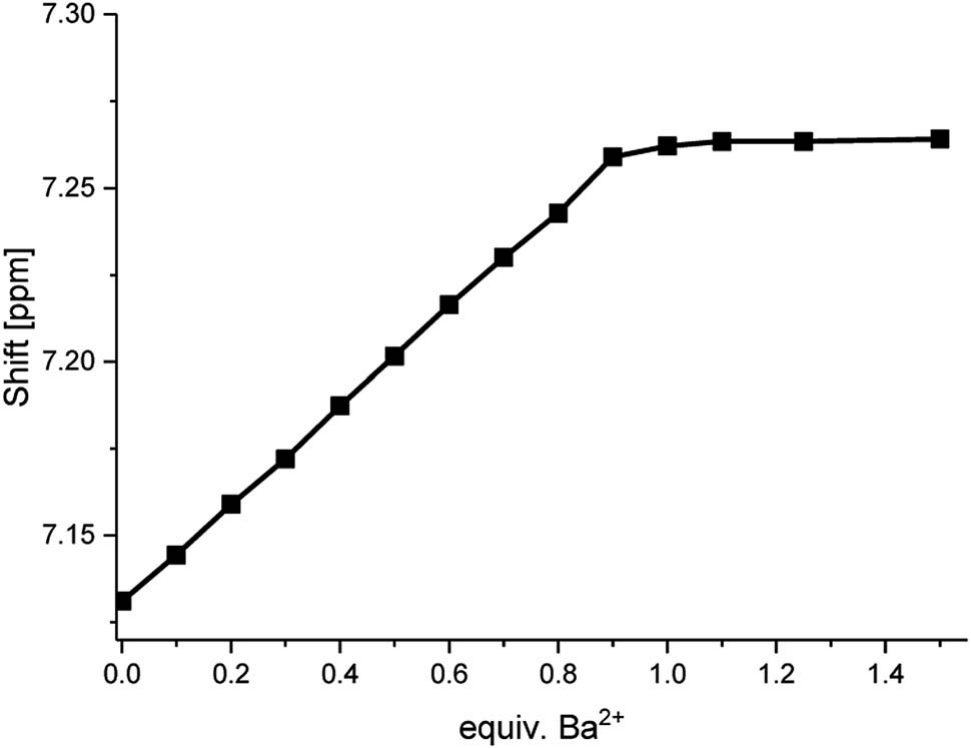
Shifts for the ^1^H NMR H4-signal of compound 1 at different Ba(ClO_4_)_2_ concentrations measured in acetonitrile-d_3_.

When plotting the ^1^H NMR chemical shifts of the signal H4 against the equivalents of Ba^2+^ (Fig. 6), a change of the slope is obtained at the ratio of 1:1, indicating the formation of a 1:1-complex. The obtained data for the shift of the signal H4 was evaluated by using the WinEQNMR2 software,^47^ calculating a stability constant of 4.6 ± 0.4.

Comparable plots and calculations were obtained for titrations with Sr^2+^ and Pb^2+^ (see ESI† and Table 3). No changes in the chemical shifts were observed when using the perchlorates of Na^+^ and Bu_4_N^+^, indicating that ligand 1 shows a size selectivity for the cations of interest.

**Table 3:** Stability constants for ligand 1 determined by NMR and UV/Vis titration in acetonitrile

Method	log *K* for the selected cations				
	Pb^2+^	Sr^2+^	Ba^2+^	Na^+^	Bu_4_N^+^
^1^H NMR	5.5 ± 0.2	4.3 ± 0.2	4.6 ± 0.4	—	—
UV/Vis	6.36 ± 0.05	4.07 ± 0.08	4.66 ± 0.02	—	—

**Fig. 7:**
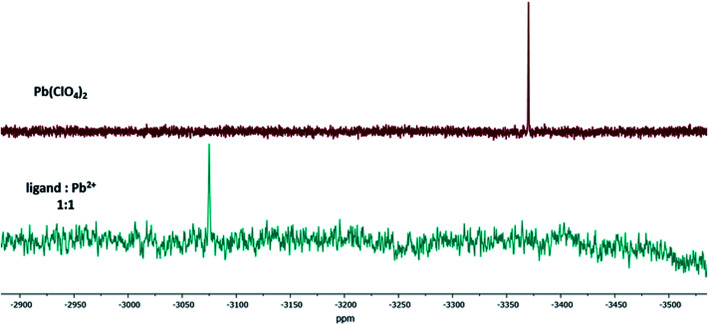
^207^Pb NMR spectra of Pb(ClO_4_)_2_ and the 1:1-Pb^2+^-complex of ligand 1 in acetonitrile-d_3_.

Additionally, the formation of the 1:1-Pb^2+^-calix-complex Pb-1 was confirmed by ^207^Pb NMR. The isotope lead-207 (natural abundance: 22.6%) has a medium sensitivity NMR spin-½ nucleus.^48,49^ For this experiment, lead(ii) perchlorate trihydrate was dissolved in acetonitrile-d_3_ and titrated with ligand 1. The difference between the ^207^Pb signal of Pb(ClO_4_)_2_ and Pb-1 resulted in a chemical shift of Δ*δ* = 296 ppm (Fig. 7). Since the trihydrate salt was used, it has to be assumed that upon complexation, the hydration shell of Pb^2+^ was released to interact with the calix 1. This exchange resulted in a downfield shifted signal.

### UV/Vis titration studies

**Fig. 8:**
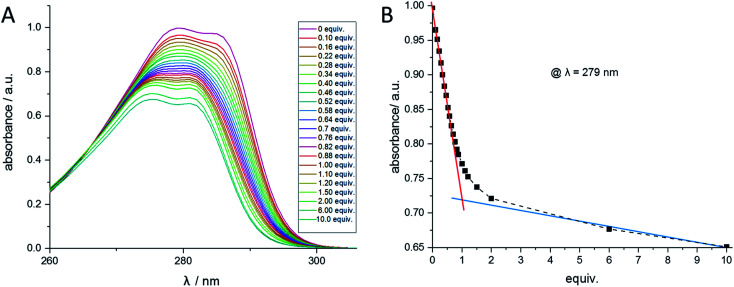
(A) UV/Vis spectra of compound 1 at different Ba(ClO_4_)_2_ concentrations measured in acetonitrile. (B) Absorption at the wavelength 279 nm of compound 1 at different Ba(ClO_4_)_2_ concentrations.

Analogous to the recently published literature,^50^ UV/Vis titrations have been performed to verify the determined stability constants. Similar to the NMR titration method, the calix ligand 1 was dissolved in acetonitrile, and aliquots of the regarding perchlorate salt dissolved in acetonitrile were added. The titration of ligand 1 with Ba^2+^ is shown in Fig. 8A. When plotting the wavelength with the maximal absorption (*λ* = 279 nm) against the equivalents of Ba^2+^, Fig. 8B is obtained, confirming the 1:1-complex Ba-1. The titrations of 1 with Sr^2+^, Pb^2+^, Na^+^, and Bu_4_N^+^ were carried out comparably (see ESI†) and led to 1:1-complexes of Sr^2+^ and Pb^2+^. No change in absorption was observed with Na^+^ and Bu_4_N^+^. The calculation of the stability constants was accomplished using the HypSpec 1.1.18 program. The results are in accordance with the values received from the NMR method and can be found in Table 3.

## Conclusion

Titration experiments with the perchlorate salts of Ba^2+^, Sr^2+^, Pb^2+^, Na^+^, and Bu_4_N^+^ and the ligand calix[4]arene-1,3-crown-6 (1) have been performed and the stability constants were determined by NMR measurements in acetonitrile-d_3_ as well as by UV/Vis measurements in acetonitrile revealing log *K* values for all divalent metals of >4 (Table 3). It was found that the stability of these complexes increase in the order of strontium, barium, and lead. No complexation was observed for Na^+^ and Bu_4_N^+^. Thus, high selectivity of the heavy alkaline earth metal ions and of Pb^2+^ was found over the control cations Na^+^ and Bu_4_N^+^. The log *K* values of both methods are comparable under the given conditions. However, both of these methods were performed close to their limits of validation,^51^ therefore, values >5 may show the right tendency but are not of high accuracy. The limits of these methods are related to the log *K* value itself and to the concentration of the host and guest molecule that is demanded to adequately detect ^1^H NMR signals or the UV/Vis absorption, respectively.^52,53^

The tendency that Ba^2+^ is showing a higher log *K* than Sr^2+^, the cation with the smaller radius, suggest that radium might provide even higher stability. Though, alternative methods have to be developed to determine higher log *K* values and to test for the radium stability as well.

## Experimental section

### General

^1^H NMR spectra were recorded on an Agilent DD2-400 MHz NMR spectrometer with ProbeOne at 298 K. Chemical shifts of the spectra were reported in parts per million (ppm) using TMS as an internal standard. All ^207^Pb spectra were recorded at a frequency of 125.1 MHz on an Agilent DD2-600 MHz NMR spectrometer with ProbeOne at 298 K using a 90° pulse width of 6.0 μs, a 0.157 s acquisition time, and a 0.6 s delay time. A 1.0 M Pb(NO_3_)_2_ solution (natural) was used as an external standard (*δ* = −2965 ppm, D_2_O, 25 °C; relative to PbMe_4_). Calix[4]arene (abcr, 99%), pentaethylene glycol di(*p*-toluenesulfonate) (Alfa Aesar, 95%), potassium carbonate anhydrous (Acros, 99+%), acetonitrile (Fisher Scientific, HPLC grade), dichloromethane (Fisher Scientific, HPLC-grade), hydrochloric acid (Merck, 37%), and sodium sulfate anhydrous (Alfa Aesar, 99%) were used as obtained for the synthesis of ligand 1. Preparative column chromatography was carried out with silica gel 60 (Merck, particle size 0.040–0.063 mm), petroleum ether (Fisher Scientific, bp 40–60 °C, analytical reagent grade), and ethyl acetate (Fisher Scientific, HPLC grade). For the preparation of the complexes, barium perchlorate (Alfa Aesar, 99%), sodium perchlorate (Alfa Aesar, 98%), and strontium perchlorate (abcr, 99.9%) were stored at room temperature under vacuum and used without further purification. Lead(ii) perchlorate trihydrate (abcr, 97%) was used as obtained. The solvents used for NMR measurements were purchased from Deutero GmbH.

### Synthesis of calix[4]arene-1,3-crown-6 (1)

The synthesis of 1 was done according to the procedure of the respective *tert*-butyl derivative.^39^ Briefly, a suspension of calix[4]arene (1.00 g, 1.54 mmol), pentaethylene glycol di(*p*-toluenesulfonate) (924 mg, 1.69 mmol) and K_2_CO_3_ (256 mg, 1.85 mmol) in acetonitrile (100 mL) was refluxed under argon for 7 days. After cooling to rt, the solvent was removed and dichloromethane (50 mL) was added. The suspension was washed with 10% HCl (2 × 50 mL) and water (1 × 50 mL). The organic layer was dried over Na_2_SO_4_. After evaporation of dichloromethane, the crude mixture was purified by column chromatography (silica gel, petroleum ether/ethyl acetate, gradient: 0 → 60%). The product was obtained as a colorless solid (580 mg, 50%). Analyses were in accordance with the previously published literature.^38^

### ^1^H NMR titration measurements

A solution of 1 was prepared in acetonitrile-d_6_ (2.0 × 10^−3^ M) and 1.0 mL was pipetted in an NMR tube. The sample was referenced to the residual solvent signal. Then, the complexation of cations with 1 was studied. A 0.1 M solution of the metal perchlorate was prepared in the same solvent. Next, stepwise portions (2 μL) of the respective perchlorate solution were added into the NMR tube containing the ligand, and after extensive mixing, the complexation-induced shifts were recorded. At a ligand : metal ratio of 2 : 3, 30 μL portions of a 1.0 M perchlorate salt solution were used, and stepwise additions were continued until a ligand : metal ratio of 1 : 6 was reached to exclude the formation of a complex with another stoichiometry. The displacements of selected ^1^H NMR signals of ligand 1 upon addition of the perchlorate salt were used to calculate the complex stability constants. All calculations were performed using the WinEQNMR2 software.^47^ The advised range for the data input covers the addition of metal to ligand from 0.1 to 0.9 equivalents. This instruction was followed and 9 points in this range were measured (steps of 0.1 equiv.) and used for the calculation. The formation of a 1:1 complex was proven by plotting the changes of selected signals against the cation to chelate ratio, observing the change of the slope at a ligand : metal ratio of 1 : 1.

### ^207^Pb NMR titration measurements

A solution of lead(ii) perchlorate trihydrate in acetonitrile-d_3_ (5.0 × 10^−2^ M) and a solution of ligand 1 in acetonitrile-d_3_ (1.0 × 10^−1^ M) were prepared. A 1.0 mL aliquot of the lead solution was pipetted in an NMR tube. The sample was measured and the ^207^Pb NMR signal determined. Next, stepwise portions of ligand 1 (40 μL, 0.08 equiv.) were added into the NMR tube containing the lead solution, and the spectra were recorded after extensive mixing.

### UV titration measurements

A solution of ligand 1 was prepared in acetonitrile. The concentration range was chosen to be 0.15 mM, depending on the absorption maximum of the compound. This solution (2.5 mL) was pipetted into a quartz cuvette of 1 cm optical path length. The titration was performed by stepwise addition of a 15 mM Ba(ClO_4_)_2_ solution in acetonitrile. The absorption spectra were measured in the range of 250 to 400 nm. The calculation of the stability constants was accomplished using the HypSpec 1.1.18 program.^54^

### X-ray structure determination

Diffraction data were collected using a Bruker Nonius Apex Kappa-II CCD diffractometer with graphite-monochromated MoKα radiation (*λ* = 0.71073 Å). The measurements were performed at −150 °C. The structure was solved by direct methods and refined against *F*^2^ by full-matrix least-squares using the program suites from G. M. Sheldrick. All non-hydrogen atoms were refined anisotropically; all hydrogen atoms were placed in geometrically calculated positions and refined by using riding models.^55–57^ CCDC 1950592 contains the supplementary crystallographic data for the title compound.†

## Conflicts of interest

There are no conflicts to declare.

## Supplementary Material

RA-009-C9RA07293D-s001

RA-009-C9RA07293D-s002
