# Supplementary material for: A comparative evaluation of calix[4]arene-1,3-crown-6 as a ligand for selected divalent cations of radiopharmaceutical interest
Source: RSC Adv. 2019 Oct 10;9(55):32357–66. doi: 10.1039/c9ra07293d (PMC9072984; doi:10.1039/c9ra07293d)
Supplement: RA-009-C9RA07293D-s001 [file RA-009-C9RA07293D-s001.pdf]

Electronic Supplementary Material (ESI) for RSC Advances.
This journal is © The Royal Society of Chemistry 2019

Supporting Information for

A comparative evaluation of calix[4]arene-1,3-crown-6 as a ligand for selected divalent cations of radiopharmaceutical interest

David Bauer,[a,b] Markus Blumberg,[a,b] Martin Köckerling,[c] and Constantin Mamat[a,b*]

a) *Institut für Radiopharmazeutische Krebsforschung, Helmholtz-Zentrum Dresden-Rossendorf, Bautzner Landstraße 400, D-013228 Dresden, Germany*

b) *Fakultät Chemie und Lebensmittelchemie, TU Dresden, D-01062 Dresden, Germany*

c) *Institut für Chemie – Anorganische Festkörperchemie, Universität Rostock, Albert-Einstein-Straße 3a, D-18059 Rostock, Germany*

Table of Contents

NMR titration experiment of calix **1** with Sr^{2+} S2

NMR titration experiment of calix **1** with Ba^{2+} S3

NMR titration experiment of calix **1** with Pb^{2+} S4

NMR titration plots of calix **1** with Ba^{2+}, Sr^{2+}, and Pb^{2+} S5

UV/Vis titration experiment of calix **1** with Ba^{2+}, Sr^{2+}, Pb^{2+} S6

UV/Vis titration plots of calix **1** with Ba^{2+}, Sr^{2+}, and Pb^{2+} S7

Crystal data for ligand **1** S8

NMR titration experiment of calix **1** with Sr^{2+}

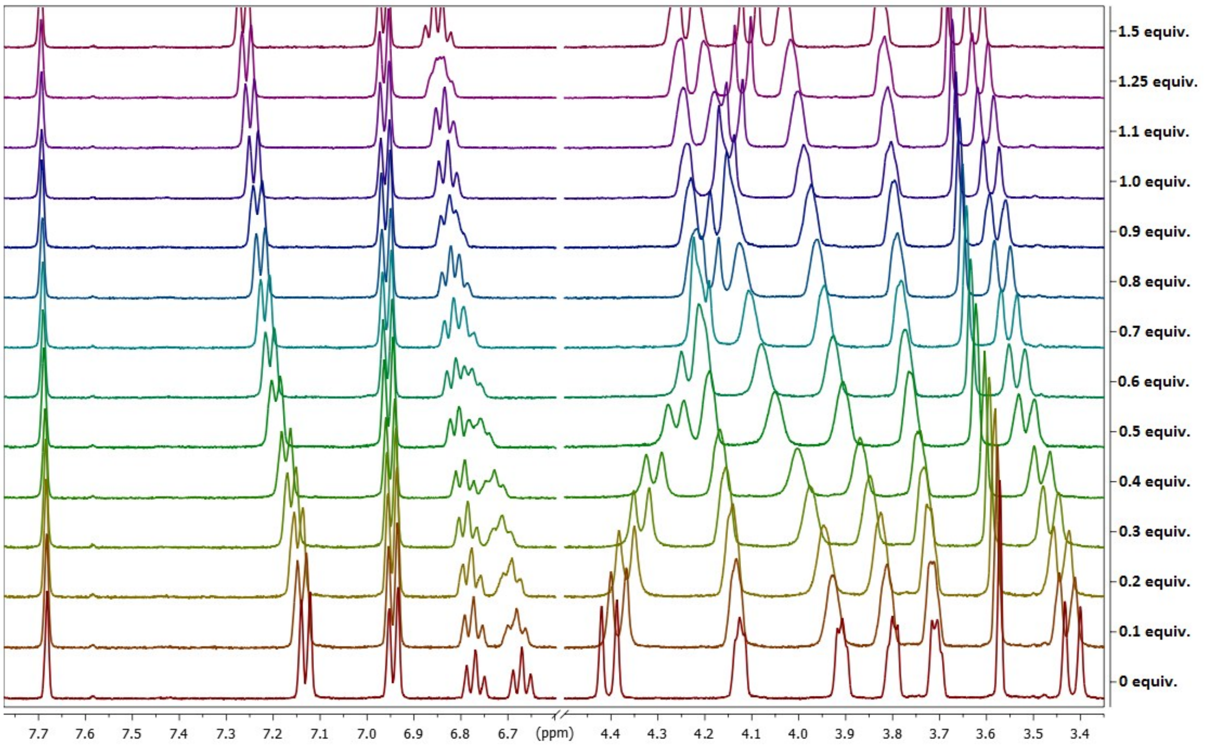

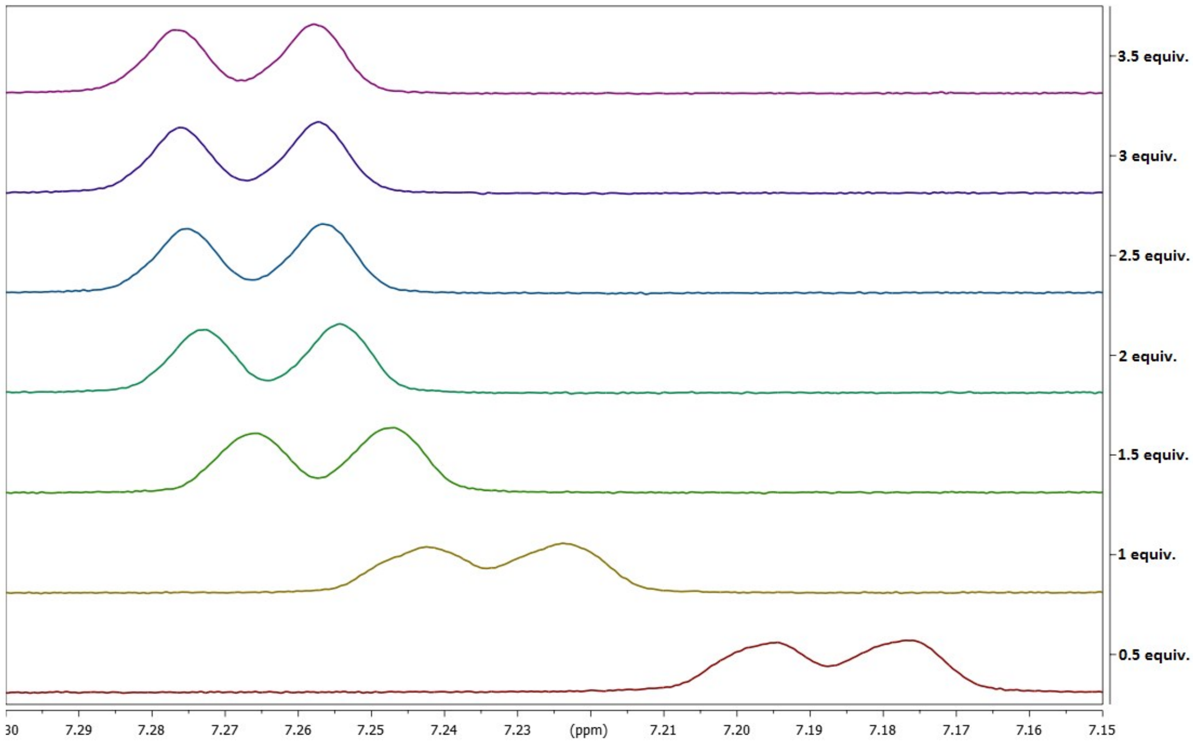

NMR titration experiment of calix **1** with Ba^{2+}

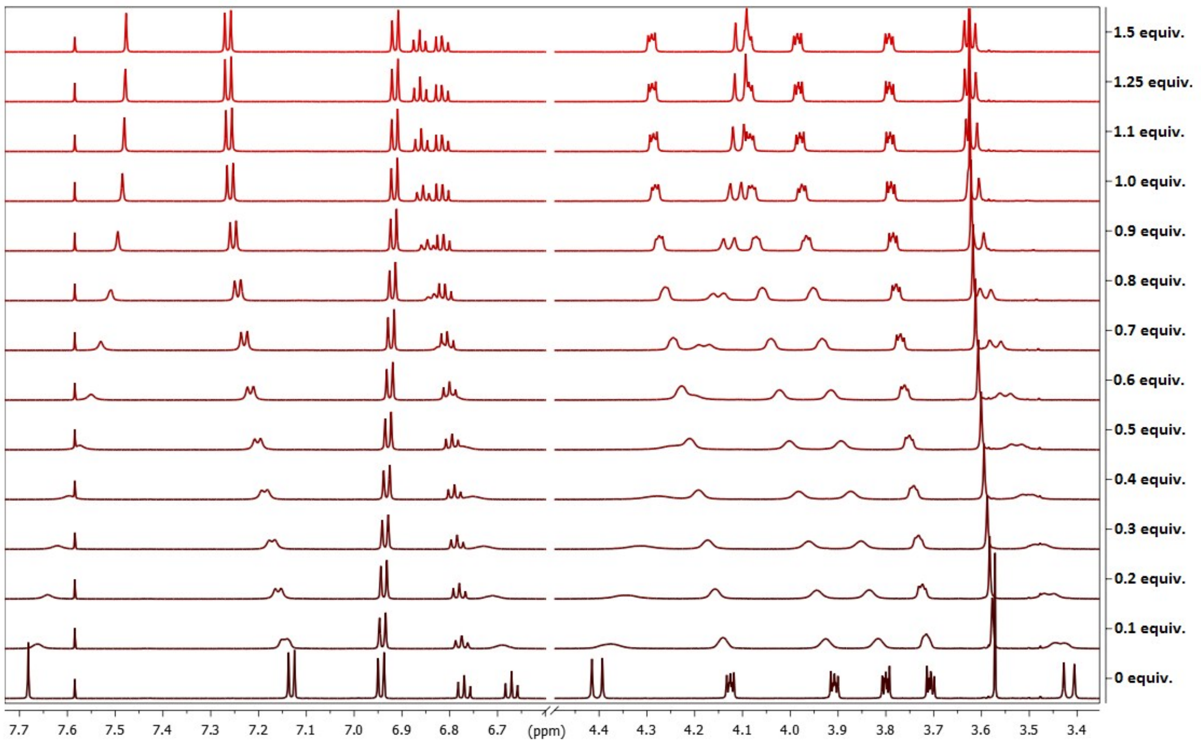

NMR titration experiment of calix **1** with Pb^{2+}

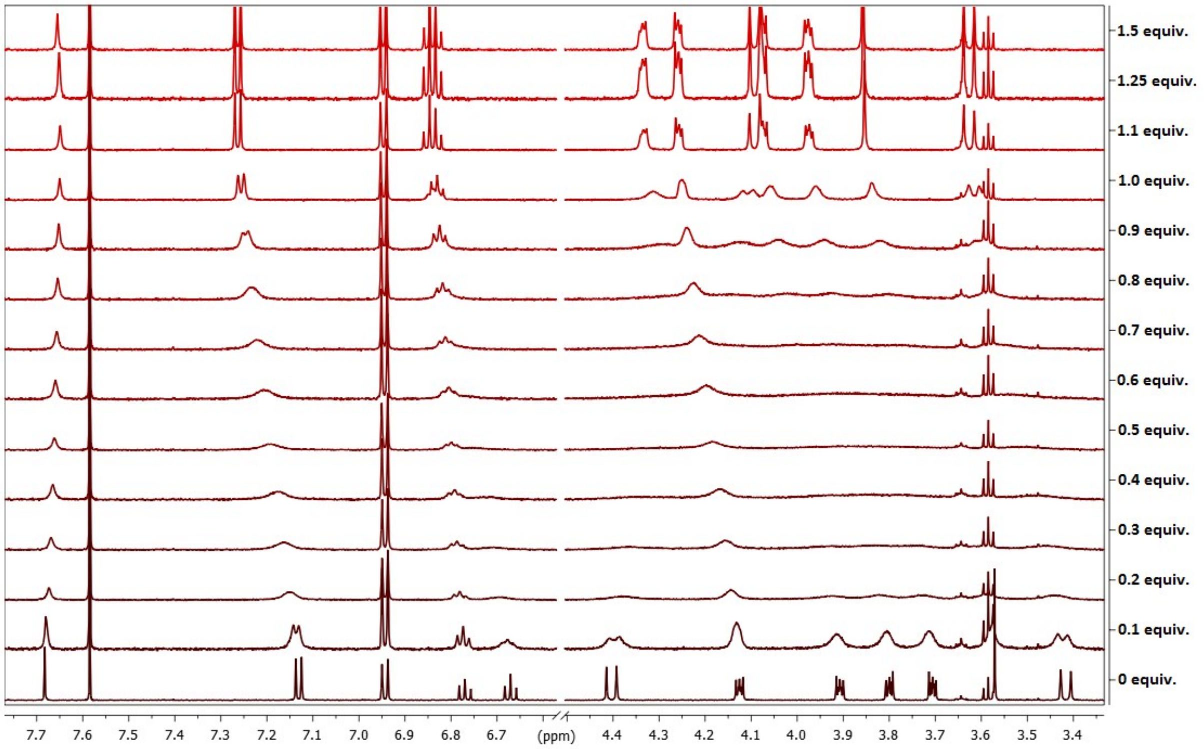

NMR titration plots of calix **1** with Ba^{2+}, Sr^{2+}, and Pb^{2+}

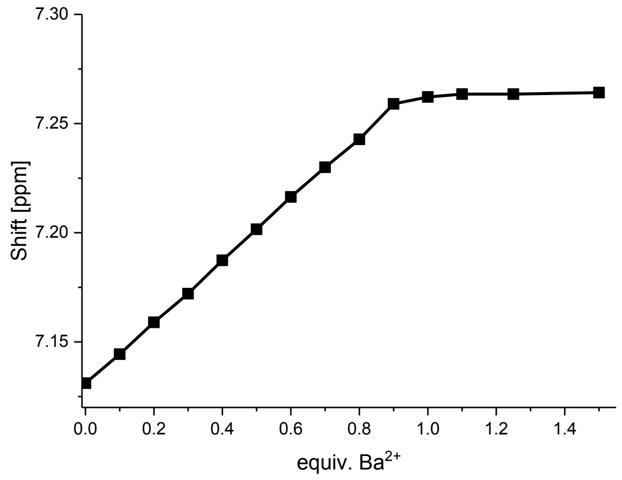

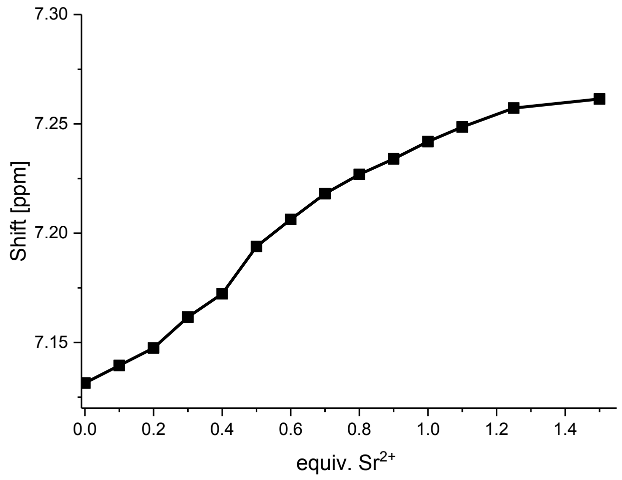

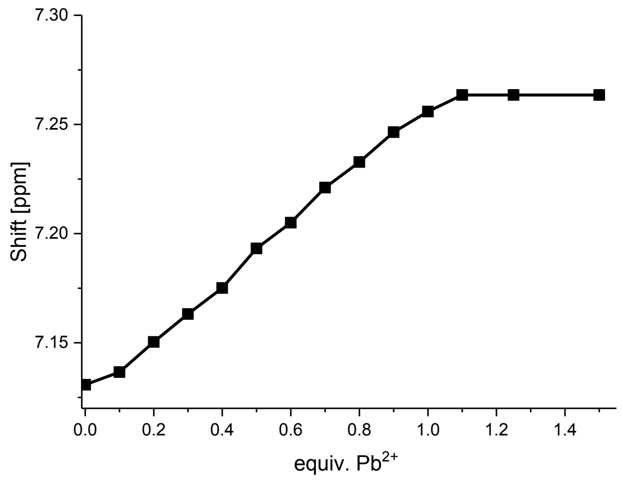

UV/Vis titration experiment of calix **1** with Ba^{2+}, Sr^{2+}, Pb^{2+}

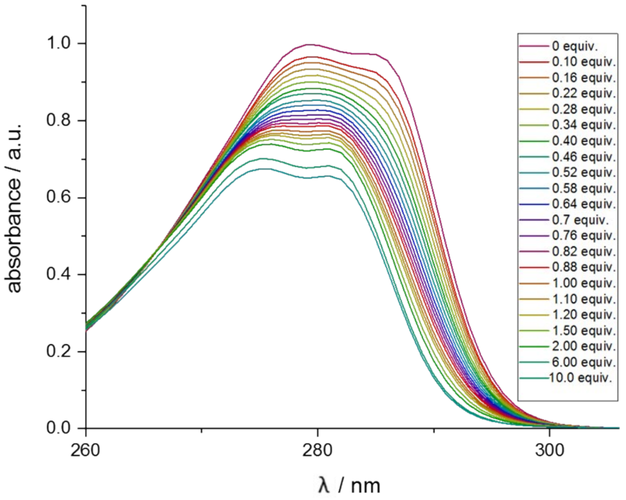

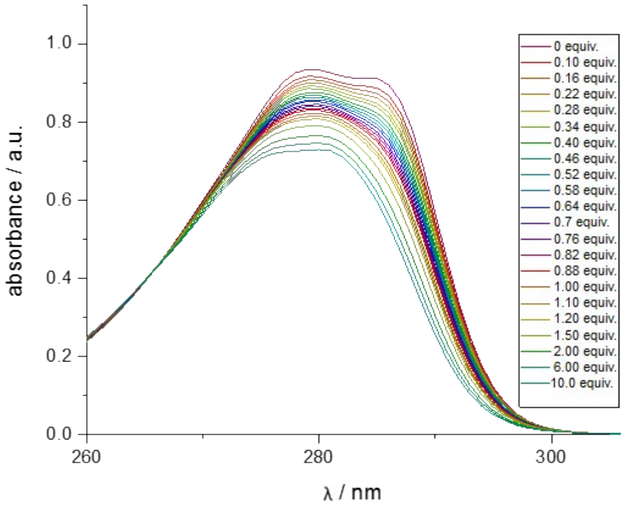

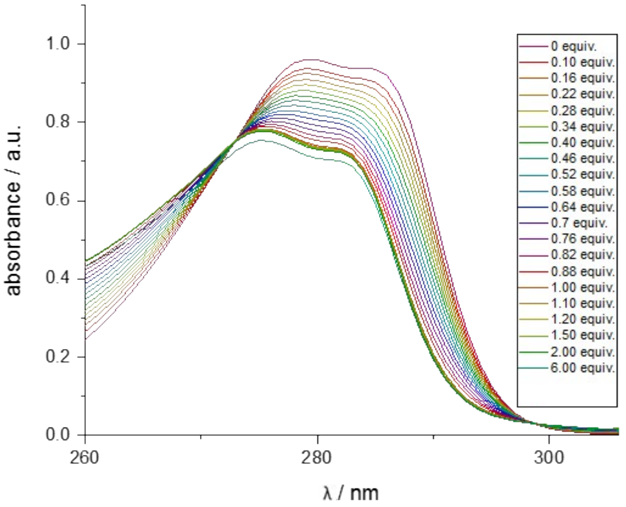

UV/Vis titration plots of calix ligand **1** with Ba^{2+}, Sr^{2+}, and Pb^{2+}

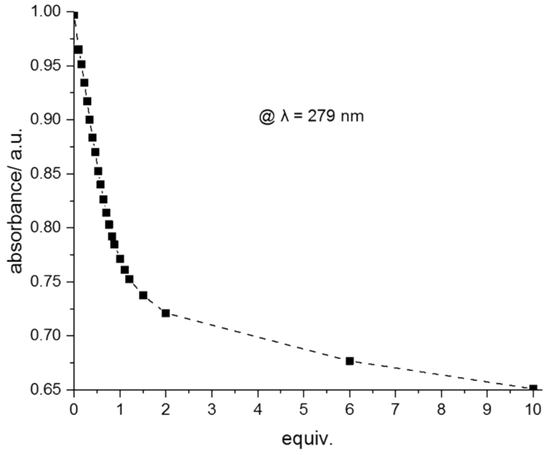

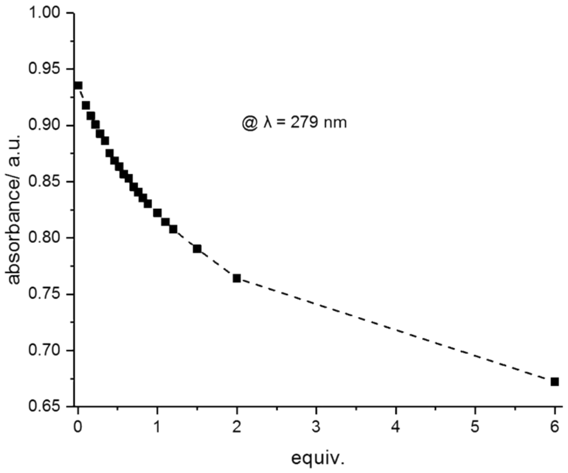

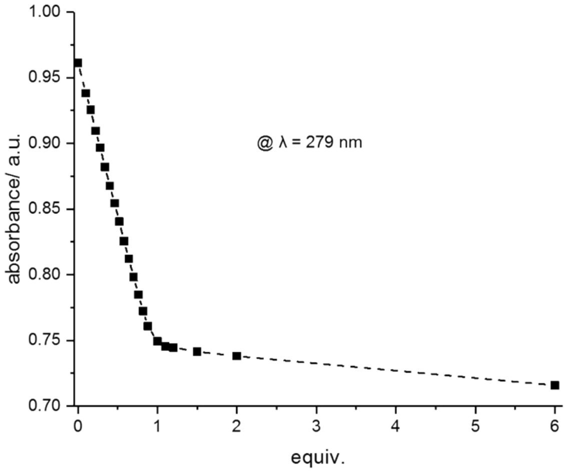

Crystal data for ligand **1**

**Crystal data**	
**Chemical formula**	$C_{78}H_{86}Cl_4O_{16}$
***M*r**	1421.26
**Crystal system, space group**	Monoclinic, *P*2/*n*
**Temperature (K)**	123
***a*, *b*, *c* (Å)**	10.0307 (3), 14.0336 (5), 12.5626 (4)
**β (°)**	100.105 (1)
**V (Å^3)**	1741.0 (1)
Z	1
**Radiation type**	Mo *Kα*
**μ (mm^{-1})**	0.24
**Crystal size (mm)**	0.15 × 0.13 × 0.13
**Data collection**	
**Diffractometer**	Bruker *APEX*-II CCD
**Absorption correction**	Multi-scan Bruker-Nonius *SADABS*
**No. of measured, independent and observed [$I > 2\sigma(I)$] reflections**	22332, 6567, 5055
**R_{int}**	0.035
**$(\sin \vartheta/\lambda)_{max}$ (Å^{-1})**	0.770
**Refinement**	
**$R[F^2 > 2\sigma(F^2)]$, $wR(F^2)$, S**	0.046, 0.125, 1.03
**No. of reflections**	6567
**No. of parameters**	264
**H-atom treatment**	H-atom parameters constrained
**$\Delta\rho_{max}$, $\Delta\rho_{min}$ (e Å^{-3})**	0.45, −0.36

Computer programs: Bruker *APEX2*, Bruker *SAINT*, *SHELXS2014* (Sheldrick, 2014), *SHELXL2014* (Sheldrick, 2014), Bruker *SHELXTL*.
